# Innovative Use of Olive, Winery and Cheese Waste By-Products as Functional Ingredients in Broiler Nutrition

**DOI:** 10.3390/vetsci9060290

**Published:** 2022-06-12

**Authors:** Eleftherios Bonos, Ioannis Skoufos, Konstantinos Petrotos, Ioannis Giavasis, Chrysanthi Mitsagga, Konstantina Fotou, Konstantina Vasilopoulou, Ilias Giannenas, Evangelia Gouva, Anastasios Tsinas, Angela Gabriella D’Alessandro, Angela Cardinali, Athina Tzora

**Affiliations:** 1 Laboratory of Animal Science, Nutrition and Biotechnology, Department of Agriculture, University of Ioannina, 47100 Arta, Greece; ebonos@uoi.gr (E.B.); egouva@uoi.gr (E.G.); 2 Laboratory of Food and Biosystems Engineering, Department of Agrotechnology, School of Agricultural Sciences, Geopolis, University of Thessaly, 41500 Larisa, Greece; petrotos@uth.gr; 3 Laboratory of Biotechnology and Applied Microbiology, Department of Food Science and Nutrition, School of Agricultural Sciences, University of Thessaly, End of N. Temponera Str., 43100 Karditsa, Greece; igiavasis@uth.gr (I.G.); cmitsanga@uth.gr (C.M.); 4 Laboratory of Animal Health, Food Hygiene and Quality, Department of Agriculture, University of Ioannina, 47100 Arta, Greece; kfotou@uoi.gr (K.F.); actsinas@uoi.gr (A.T.); tzora@uoi.gr (A.T.); 5 Laboratory of Nutrition, Faculty of Veterinary Medicine, Aristotle University of Thessaloniki, 54124 Thessaloniki, Greece; kwnstantw@hotmail.com (K.V.); igiannenas@vet.auth.gr (I.G.); 6 Department of Agro-Environmental and Territorial Sciences, University of Bari, Via Amendola 165/A, 70126 Bari, Italy; angelagabriella.dalessandro@uniba.it; 7 National Research Council—Institute of Science of Food Production, Via Amendola 122/O, 70126 Bari, Italy; angela.cardinali@ispa.cnr.it

**Keywords:** broilers, bioactive silage, antioxidant status, microbiology, intestine, meat

## Abstract

The purpose of this study was to evaluate the dietary use of novel silage that was created by combining three agro-industrial wastes produced in bulk, i.e., olive mill wastewater, grape pomace, and deproteinized feta cheese whey, in the diets of broiler chickens. A total of 216 one-day-old male Ross-308 chicks were randomly allocated to three treatment groups with six replications (12 chicks per pen). Three isocaloric and isonitrogenous diets were formulated to include the examined silage at 0%, 5%, or 10%. Commercial breeding and management procedures were employed throughout the trial. At the end of the trial (day 35), tissue samples were collected for analysis. Feeding 10% silage resulted in increased (*p* ≤ 0.001) final body weight (*p* ≤ 0.001) and feed intake. Jejunum and cecum microflora, as well as breast and thigh meat microflora, were modified (*p* ≤ 0.05) by the dietary inclusion. Thigh meat oxidative stability was improved (*p* < 0.01) by the silage supplementation. In addition, breast and thigh meat fatty acid profiles were different, respectively, (*p* < 0.05) in the supplemented treatments compared to the control. The examined silage was successfully tested in broiler diets with potential benefits for their performance and meat quality.

## 1. Introduction

The selection of feed has a major role in poultry production sustainability and productivity. The chicken feed industry in Europe cannot find adequate quantities of locally produced feed material and has to import from abroad. Thus, feed material availability and price can range widely, and especially in the last year, the price of important feeds such as cereals and soybean meal has risen steeply worldwide. However, in many European countries, various agro-industrial wastes are produced in large quantities that could potentially be used in the feed industry. Such wastes are, for example, olive mill wastewater solids, grape pomace solids, and whey solids. These wastes contain valuable biomass, as well as important bioactive compounds such as polyphenols, flavonoids, carotenoids, dietary fiber, and unsaturated fatty acids [1]. Due to their physicochemical properties, it is usually impractical to incorporate these wastes into feed formulations and actual production. Nevertheless, new technologies are being developed to process them into more appropriate forms. These technologies include solid substrate fermentation, ensiling and solid or slurry processing [1,2]. The development of such technologies takes into consideration the particularities of local animal production conditions that could result in applicable products for animal feed use.

Silages are semiliquid or paste products that are commonly used in animal nutrition. There are different methods of preparing silages, including chemical methods (using organic and inorganic acids), microbiological methods (using microbial cultivations as starters), and enzymatic methods (using proteolytic and fibrolytic enzymes) [3,4]. The silage undergoes fermentation which modifies both the chemical composition and the microbiota balance of the feed material and which is characterized by the proliferation of homo-fermentative and hetero-fermentative lactic acid bacteria and the transformation of simple plant carbohydrates into organic acids including lactic and acetic acids [5,6]. Agricultural by-products can be included in silage processing to lower the overall cost, but also to incorporate into the silage valuable nutrients such as antioxidants or unsaturated fatty acids. Although silages are more commonly used in ruminant nutrition, silages created with waste by-products are also under investigation in other farm animals such as chickens with promising results [7,8].

In the present work, innovative silage created previously by our team [1] by the optimized combination of three common agro-industrial wastes, olive mill wastewater solids, grape pomace solids, and feta cheese whey solids, were tested for the first time in broiler chicken diets. Data on broiler performance, health and welfare status, and meat quality parameters were evaluated.

## 2. Materials and Methods

### 2.1. Animals, Diets, and Experimental Design

This trial was carried out in accordance with the principles and regulations of the local veterinary services [9] and the authorities of the School of Agriculture of the University of Ioannina, Greece (UOI University Research Committee research registration: 60570). Throughout the trial, the birds were monitored by a veterinary surgeon.

Two hundred and sixteen, one-day-old male Ross-308 chicks (initial body weight 42.1 ± 0.4 g) were procured from PINDOS APSI hatchery (Ioannina, Greece) and housed at a commercial poultry farm in Arta (latitude 38.617°, longitude 20.767°), Epirus, Greece, during the period of November-December 2020. Each treatment group consisted of 6 replicate pens (length 1.0 m; width 1.1 m) of 12 chicks each. During the trial, commercial breeding and management procedures were employed, natural and artificial light was provided on a basis of 23 h for the first two days, 16 h from day three to day 14, and 21 h from day 15 to slaughter (day 35). Ambient temperature and humidity were controlled. All birds were vaccinated against Newcastle disease, infectious bronchitis, and infectious bursal disease (Gumboro) at the hatchery. Feed and drinking water were offered to all birds ad libitum throughout the experiment.

The design and optimization of the examined novel silage created by agro-industrial by-products are described in detail in Petrotos et al. [1]. Briefly, initially many different mixing ratios of olive mill wastewater solids, grape pomace solids, and whey solids were tested to create silages, and after fermentation, these silages were evaluated based on their chemical and microbiological criteria by using advanced mathematical modeling. The best silage was then used for this broiler chicken trial. The chemical composition [10] of this silage is presented in Table 1.

Control treatment (Silage-0%) chickens were fed commercial typical rations in mash form, based on maize and soybean meal (Table 2) that were formulated according to breeder recommendations [11]. The other two treatments were formulated to include either 5% or 10% of the examined silage (named Silage-5% and Silage-10%, respectively). To formulate these rations, the ingredient matrix data from the databases of Premier Nutrition [12] and NRC [13] were used. All three diets were formulated to be isocaloric and isonitrogenous.

Individual body weight was recorded on days 1, 15, 22, and 35. Feed consumption and mortality were recorded daily. At the end of the trial (day 35), all birds were slaughtered under commercial conditions (pre-slaughter electrical stunning, bleeding, scalding, defeathering, evisceration). From each replicate pen, 4 birds were randomly selected for meat analysis and 4 for blood and intestinal microbiological analyses and were individually marked (leg bands) for identification.

### 2.2. Gastrointestinal Tract Sampling

The abdomen of each chicken was cleaned with 70% (*v*/*v*) ethanol and skin incisions were made to give good access to the intestine. The caeca and jejunum of each bird were carefully processed as described by Yan et al. [14]. The intestinal contents and mucosa were mixed uniformly before storage.

### 2.3. Bacterial Cultivation and Bacterial Counts

For the intestinal microflora analyses, initially, 1 g of the collected samples was homogenized with 9 mL of 0.1% sterile peptone water solution. Moreover, for the meat analyses, initially, 10 g of breast or thigh meat were homogenized in Bagmixer 400 (Interscience, France) with 90 mL of sterile maximum recovery diluent (MRD, Oxoid, Basingstoke, UK). Then, for the bacterial enumeration of all samples, the Miles and Misra Plate Method (surface drop) [15] was used and each sample was diluted serially via 10-fold dilutions (from 10^−1^ to 10^−12^) using standard 96-well plates for microdilutions. Ten microliters of each dilution were inoculated on media and incubated as follows: total aerobic and anaerobic counts were determined using plate count agar (PCA, Oxoid) medium, while plates were incubated at 30 °C aerobically for 48 h and at 37 °C anaerobically for 48–72 h, respectively. MacConkey and Kanamycin aesculin azide (KAA) agar (Merck, Darmstadt, Germany), were, respectively, used for the isolation, enumeration, and identification of *Escherichia coli* and enterococci, and all plates were incubated aerobically at 37 °C for 24–48 h. De Man, Rogosa, and Sharpe (MRS) agar (Oxoid) and Tryptose sulfite cycloserine (TSC) agar (Merck) were, respectively, used for the isolation, enumeration, and identification of lactobacilli and *Clostridium* spp., while media were incubated at 37 °C for 48 h in anaerobic conditions. *Bifidobacterium* isolation and enumeration were performed on transoligosaccharide propionate agar medium (TOS, Merck) supplemented with glacial acetic acid (1%, *v*/*v*) and mupirocin (100 μL/mL) and were incubated anaerobically at 37 °C for 72 h. *Campylobacter jejuni* was isolated from campy blood-free selective medium (CCDA, Acumedia—Lab M, Lansing, MI, USA) with *Campylobacter* selective supplement under microaerophilic conditions in incubator with 10% CO_2_ at 37 °C for 72 h. *Staphylococcus aureus* and *Staphylococcus* spp. were isolated from Baird Parker agar (Oxoid) which was supplemented with egg yolk tellurite (50 mL/1 l substrate) and incubated under aerobic conditions at 37 °C for 48 h.

Bacterial counts were expressed as log_10_ colony-forming units (CFU) per 1 g wet weight sample. Typical colonies grown on media were then described and subcultured. Identification of all bacterial isolates was performed by Bruker MALDI Biotyper (Bruker Daltonik, Leipzig, Germany). Isolates and control strains on agar plates were subjected to MALDITOF MS on a Microflex LT instrument (Bruker Daltonik) as previously described [16,17,18]. Briefly, the bacterial culture on MALDI plates was overlaid with 1 μL of matrix solution containing 10 mg/mL of a-cyano-4-hydroxycinnamic acid (Sigma-Aldrich, Prague, Czech Republic) dissolved in 50% acetonitrile (Sigma-Aldrich) and 2.5% trifluoroacetic acid (Sigma-Aldrich), and then air-dried. The mass spectra were processed using the MALDI Biotyper 3.0 software package (Bruker Daltonik) containing 6903 reference spectra. Identification was performed according to the criteria recommended by the manufacturer (ID score: 1.700–1.999 probable genus identification; 2.000–2.299 secure genus identification, probable species identification; 2.300–3.000 highly probable species identification).

### 2.4. Blood Parameters Analysis

On the last day of the trial, feeds were removed 4 h prior to blood sampling. From each chicken, blood samples were collected aseptically for hematology and lipid blood analysis. Each live bird was gently removed from the pen and held by an assistant. Blood samples were obtained into lithium–heparin vials for the determination of biochemical parameters (Triglycerides, TRIG; Albumine, ALB; Alanine aminotransferase, ALT; aspartate aminotransferase, AST; Cholesterol, CHOL; Total bilirubin, TBIL; Glucose, GLU) [19]. After centrifugation, the serum samples were analyzed using the IDEXX VETTEST 8008 apparatus (IDEXX LAB, Westbrook, ME, USA) according to the manufacturer’s instructions.

### 2.5. Meat Chemical and ColorAnalyses

The birds used in the analysis of the meat chemical composition were transported and processed in a commercial slaughterhouse, according to local practices. Their carcasses were scalded at 61–65 °C for 60 s, defeathered in a rotary drum picker for 25 s and whole carcasses (head, feet, blood, without intestines) were air-chilled at 4 °C. After chilling, carcasses were weighed 24 h post-mortem. From each carcass, initially, the whole breast and the two legs (with back attached) were cut. Then, the breast meat and the thigh meat were carefully separated from skin and bones, and then they were ground using a meat grinder (Bosch, Gerlingen, Germany). Samples of 200 g of the minced meat were analyzed for moisture, crude protein, and fat content, by near infra-red spectroscopy using a FoodScan^TM^ Lab (FOSS, Hillerod, Denmark) in transmittance mode, by the reference method AOAC 2007.04 for meat and meat products [10,20].

Meat color of the breast and meat samples was analyzed with a “CAM-System 500 Chromatometer” (Lovibond, Amesbury, UK). The “L*A*B*” color space of the samples was evaluated, which represents lightness (L*), redness (A*), and yellowness (B*) values, respectively.

### 2.6. Meat Oxidative Stability Analysis

Meat total phenols were determined as described in Jang et al. [21]. Lipid oxidation status of meat samples was determined as described by Ahn et al. [22] with minor modifications, using a spectrophotometer (UV 1700 PharmaSpec, Shimadzu, Kyoto, Japan) set at 532 nm. Lipid oxidation was determined as the 2-thiobarbituric acid-reactive substances (TBARS) value, expressed as mg of malondialdehyde (MDA)/kg of meat.

### 2.7. Meat Fatty Acid Analysis

For the breast and thigh meat fatty acid analysis, samples were processed as recommended by O’Fallon et al. [23]. Then, separation and quantification of the methyl esters were performed as described in Skoufos et al. [24] using a TraceGC (Model K07332, Thermofinigan, Thermoquest, Milan, Italy) equipped with a flame ionization detector.

### 2.8. Statistical Analysis

The basic study design was RCB (random complete block design) and the replication (pen) was considered the experimental unit. Experimental data were analyzed by one-way analysis of variance (one-way ANOVA) of the SPSS statistical package (version 20.0) was applied [25]. Microbiology data were log-transformed (log10) prior to analysis. Data homogeneity was tested using Levene’s test. Tukey’s test was used for post hoc comparisons between the three treatments. Significance level for all tests was set at 5% (*p* ≤ 0.05).

## 3. Results

### 3.1. Performance Parameters

The effects of the dietary supplementation with the silage on broiler performance are presented in Table 3. Treatment Silage-10% had increased final body weight (*p* = 0.001), increased overall body weight gain (*p* = 0.001) and increased overall feed intake (*p* < 0.001) compared to the other treatments. Moreover, treatments Silage-5% and Silage-10% had higher overall feed conversion ratios (*p* = 0.011) compared to treatment Silage-0%.

### 3.2. Intestinal Microflora

The intestinal microflora was affected by the silage supplementation (Table 4). In the jejunum, the supplemented treatments Silage-5% and Silage-10% had higher total anaerobes (*p* = 0.001), lower Enterobacteriaceae (*p* = 0.001), lower enterococci (*p* < 0.001) and higher bifidobacteria (*p* = 0.008), compared to the control treatment Silage-0%. In the cecum, treatment Silage-10% had higher total anaerobes (*p* = 0.041) compared to treatment Silage-5%. Furthermore, treatment Silage-10% had lower Enterobacteriaceae (*p* = 0.002) and higher bifidobacteria (*p* = 0.050) compared to treatment Silage-0%. Additionally, treatment Silage-10% had higher lactobacilli counts (*p* = 0.001) compared to treatments Silage-0% and Silage-5%.

### 3.3. Blood Parameters

Table 5 shows the results of the examined broiler blood parameters. Treatments Silage-5% and Silage 10% had lower (*p* = 0.006) blood triglycerides (TRIG), compared to treatment Silage-0%. Treatment Silage-10% had lower (*p* = 0.020) blood cholesterol (CHOL) compared to treatment Silage-0%. In addition, treatment Silage-10% had higher (*p* = 0.001) blood glucose (GLU) compared to the other two treatments.

### 3.4. Meat Analysis

As shown in Table 6, the breast and thigh meat chemical composition did not differ (*p* ≥ 0.05) between the treatments regarding fat, moisture, protein, collagen, and ash. However, the determination of the meat color showed that the breast meat of treatment Silage-10% had a higher (*p* = 0.008) B* value, compared to treatment Silage-5%. However, the other examined parameters did not differ (*p* ≥ 0.05) between the treatments.

The breast and thigh meat microbial analyses is given in Table 7. In the breast meat, treatment Silage-10% had lower (*p* = 0.042) total microbes compared to the other two treatments. Furthermore, treatments Silage-5% and Silage-10% had lower (*p* = 0.001) *Escherichia coli*, lower (*p* = 0.003) *Clostridium* spp. and lower (*p* < 0.001) *Campylobacter jejuni*, compared to the control treatment Silage-0%. In the thigh meat, treatments Silage-5% and Silage-10% had lower (*p* = 0.008) *Escherichia coli*, compared to treatment Silage-0%. Moreover, treatment Silage-10% had lower (*p* = 0.001) *Campylobacter jejuni* counts compared to the other two treatments.

The oxidative stability analysis (Table 8) of the breast and thigh meat showed that Silage-10% had lower thigh meat TBARS (*p* = 0.030), compared to the other two treatments. The other examined parameters (meat MDA and meat phenols) did not differ (*p* ≥ 0.05) between the treatments.

Fatty acid analysis of the breast meat (Table 9) identified some differences between the three treatments. Myristoleic acid was lowest (*p* = 0.001) in Silage-10% compared to the other two treatments; palmitic acid was lower (*p* = 0.003) in Silage-10% compared to the other two treatments; palmitoleic acid was lower (*p* = 0.006) in Silage-5% and Silage-10% compared to Silage-0%; stearic acid was higher (*p* = 0.008) in Silage-10% compared to Silage-0%; elaidic acid was highest (*p* < 0.001) in Silage-10% compared to the other two treatments; γ-linolenic acid was lower (*p* = 0.024) in Silage-5% compared to Silage-0%; cis-11.14-eicossadienoic was higher (*p* = 0.021) in Silage-10% compared to Silage-0%; arachidonic acid was higher (*p* = 0.002) in Silage-5% and Silage-10% compared to Silage-0%; cis-4.7.10.13.16.19-docosahexaenoic was highest (*p* = 0.030) in Silage-5% and Silage-10% compared to Silage-0%; total monounsaturated fatty acids were lower (*p* = 0.035) in Silage-10% compared to Silage-10%.

Fatty acid analysis of the thigh meat (Table 10) also identified some differences between the three treatments. Cis-10-heptadecenoic was higher (*p* = 0.010) in Silage-5% and Silage-10% compared to Silage-0%; arachidic acid was higher (*p* = 0.006) in Silage-5% and Silage-10% compared to Silage-0%; α-linolenic acid was higher (*p* = 0.032) in Silage-10% compared to Silage-0%; cis-11.14-eicossadienoic acid was higher (*p* = 0.019) in Silage-5% and Silage-10% compared to Silage-0%; arachidonic acid was lowest (*p* = 0.025) in Silage-5% and Silage-10% compared to Silage-0%; nervonic acid was lower (*p* = 0.018) in Silage-5% and Silage-10% compared to Silage-0%; cis-4.7.10.13.16.19-docosahexaenoic was lowest (*p* = 0.028) in Silage-5% and Silage-10% compared to Silage-0%; total saturated fatty acids were lower (*p* = 0.028) in Silage-10% compared to Silage-0%; total unsaturated fatty acids were higher (*p* = 0.029) in Silage-10% compared to Silage-0%.

## 4. Discussion

A review of the international scientific literature shows large efforts in recent years concerning the use of agro-industrial food wastes as raw materials [1,26]. Although there is a great variety of tested material and processing methods, to our knowledge the examined combination of olive mill wastewater solids, grape pomace solids, and feta cheese whey solids was tested in the current study for the first time in broiler chicken diets.

Silages are commonly used in ruminant nutrition. Their use in poultry and especially broilers is not so common, probably due to the fact that most examined silages contain forages with average or high amounts of insoluble fiber for which the digestive tract of chicken cannot produce the enzymes necessary to digest [8]. However, it appears that moderate amounts of insoluble fiber can have positive effects on nutrient digestibility and the overall health of chicken [27]. Negative effects of silage and haylage have been reported on chicken body weight gain and feed intake, compared to chickens or hens fed only pellets with concentrate feeds [8,28]. Other researchers reported promising results [29]. In our case, growth was improved by the silage supplementation, although feed intake was increased, and feed conversion ratio was higher for the group that was fed 10% silage. It is possible that differences in performance results can be explained by the sensitivity of fast-growing chicken to feed texture and structure or different methods of incorporation of the silage in the pellet [30]. It has been suggested that slower growth chicken breeds, such as those used in organic or small scale-farms, could benefit more from the inclusion of silages in the overall diets [8].

It is well known that the welfare and productivity of broiler chickens are strongly influenced by their intestinal microbiome. This microbiome shows great variability in the number of microbial species and overall counts that are further affected by several parameters such as the age of the bird, the health condition of the gastrointestinal tract, and the use of various feed components (Oakley et al., 2014; Petricevic et al., 2018). Avian gastrointestinal balance is imperative for efficient digestion, nutrient absorption, and immune response to pathogens. This balance is a dynamic phenomenon depending on various parameters and large population shifts can take place due to infection or dietary imbalances (Oakley et al., 2014; McDonald et al., 2017; Tzora et al., 2017). In our experiment, microbial population analysis by MALDI-TOF MS showed that the silage supplementation increased the lactobacilli and bifidobacteria populations in the cecum while at the same time lowered the Enterobacteriaceae populations. Lactobacilli and bifidobacteria taxa are generally considered beneficial for chickens, whereas many Enterobacteriacea species can be considered potential pathogens (Liu et al., 2017; Tzora et al., 2021). Moreover, in our study microbiological analysis of breast and thigh meat showed that the meat of the supplemented treatments had significantly lower counts of pathogenic bacteria such as *E. coli*, *Clostridium* spp., and *C. jejuni*, which show a potential beneficial link between changes in the gastrointestinal tract and overall health status of the birds and the produced chicken meat. The reduction of bacterial counts in the poultry meat is very important for the hygienic quality of this product since testing of carcass contamination by fecal microorganisms is considered one of the most important control points in hazard analysis systems [31].

In this experiment, a statistically significant antioxidant effect was seen on thigh meat of poultry that were fed 10% silage. The elevated amount of polyphenols in the tested silage could act as antioxidant agents that can counteract reactive oxygen species (ROS) and protect chicken cells and tissues from oxidative damage to the membrane layer [32,33]. Numerous published works have linked the beneficial properties of dietary plant antioxidants as defense mechanisms against lipid oxidation of the produced poultry meat [34,35,36,37]. For example, Gerasopoulos et al. [7] found that broilers fed rations supplemented with maize silage produced with the inclusion of olive mill wastewaters retentate or permeate showed significantly lower protein oxidation and lipid peroxidation levels and higher total antioxidant capacity in blood and meat tissues compared to a negative control treatment. Another study highlighted the in vitro antioxidant ability of olive mill wastewaters, originating from Italian and Greek olive cultivars after membrane filtration processing [38]. In addition, Makri et al. [39] examined the potential antioxidant effects of a feed supplemented with grape pomace in chickens and reported that this supplementation decreased oxidative stress-induced toxic effects (lipid and protein oxidation) and improved chickens’ redox status in blood and tissues of intestinal organs.

Meat chemical composition was not affected by the dietary supplementation of the examined silage. Notably, some difference was found between the two supplemented treatments regarding the breast meat yellowness (B*). Color is an important acceptability parameter since the consumer will often reject products with colors that vary from what is expected as “normal”, while color also determines the economic value of the product [40]. However, the fatty acid profile analysis showed significant differences in the fatty acid profiles of both breast and thigh meat. It has been suggested that increasing the dietary feed content of n-3 polyunsaturated fatty acids and lowering the n-6/n-3 ratio can be beneficial for lipid metabolism in farm animals such as poultry and pigs, lowering obesity-induced inflammations and insulin resistance [41,42,43]. Moreover, diets rich in n-3 polyunsaturated fatty acids could significantly affect blood composition, lowering serum cholesterol levels and glucose [43]. In monogastric animals such as poultry and pigs, there is often a correlation between feed fatty acid composition, fat metabolism, and fat deposition in edible tissues. Dietary enrichment with polyunsaturated fatty acids such as linoleic, α-linolenic and arachidonic acids is often linked to elevated levels of these acids in the muscle and adipose tissues both through direct incorporation and modification of unsaturated fatty acids synthesis in these tissues [41,44]. The underlying mechanisms are complex, affecting the expression of lipogenic genes [45,46].

## 5. Conclusions

A recent review of the international scientific literature shows a large effort concerning the use of agro-industrial food wastes as raw materials. Ensilaging is a promising method to reprocess by-product wastes and produce low cost but high nutritional value feeds for farm animals such as chickens. Silage that was examined in this trial was created by the optimized combination of three common agro-industrial wastes, olive mill wastewater solids, grape pomace solids, and feta cheese whey solids, and was tested for the first time in broiler chicken diets with good performance results and acceptable meat quality. Further research is necessary to test this silage in other poultry diets.

## Figures and Tables

**Table 1:** Chemical analysis of the examined silage.

Chemical Analysis	
Moisture (%)	42.89
Dry matter (%)	57.11
Ash (%)	1.15
Crude fat (%)	3.21
Crude fiber (%)	2.63
Crude protein (%)	5.51
Total Ca (%)	0.05
Total P (%)	0.18
Mn (mg/kg)	16.95
Fe (mg/kg)	82.48
Cu (mg/kg)	3.21
Zn (mg/kg)	30.43

**Table 2:** Broiler chicken diets.

	Starter Feed (Days 1–21)			Finisher Feed (Days 22–35)		
Ingredients (%)	Silage-0%	Silage-5%	Silage-10%	Silage-0%	Silage-5%	Silage-10%
Maize	58.736	52.086	45.436	63.410	56.760	50.110
Innotrition Silage	0.000	5.000	10.000	0.000	5.000	10.000
Soybean meal (47% CP)	34.555	35.147	35.738	29.505	30.097	30.689
Soybean oil	2.930	3.986	5.041	3.567	4.623	5.678
Limestone	0.395	0.383	0.370	0.281	0.268	0.256
Monocalcium phosphate (22% P)	0.630	0.653	0.676	0.495	0.518	0.540
Methionine DL	0.219	0.223	0.227	0.189	0.193	0.197
Lysine HCl	0.035	0.024	0.012	0.053	0.042	0.030
Mineral and vitamin Premix *	2.500	2.500	2.500	2.500	2.500	2.500
**Total**	100.000	100.000	100.000	100.000	100.000	100.000
**Chemical analysis**						
Apparent Metabolisable Energy, kcal/kg	3050.00	3050.00	3050.00	3150.00	3150.00	3150.00
Crude Protein, %	21.50	21.50	21.50	19.50	19.50	19.50
Dry Matter, %	88.03	86.67	85.32	88.03	86.67	85.32
Ash, %	5.84	5.85	5.87	5.34	5.36	5.37
Crude Fat, %	5.62	6.55	7.48	6.33	7.26	8.19
Crude Fiber, %	2.66	2.62	2.58	2.54	2.50	2.45
ADF, %	3.12	3.08	3.04	2.96	2.92	2.87
NDF, %	8.32	8.07	7.82	8.30	8.05	7.79
Calcium, %	0.87	0.87	0.87	0.79	0.79	0.79
Total Phosphorus, %	0.70	0.70	0.70	0.65	0.65	0.65
Lysine, %	1.26	1.26	1.26	1.14	1.14	1.14
Methionine + Cystine, %	0.97	0.97	0.97	0.89	0.89	0.89

* Supplying per kg feed: 15,000 IU vitamin A, 5000 IU vitamin D3, 50 mg vitamin E, 4 mg vitamin K, 3 mg thiamine, 8 mg riboflavin, 5 mg pyridoxine, 0.016 mg vitamin B12, 60 mg niacin, 18 mg pantothenic acid, 1.5 mg folic acid, 0.2 mg biotin, 450 mg choline chloride, 100 mg Zn, 120 mg Mn, 80 mg Fe, 20 mg Cu, 1.0 mg I, 0.3 mg Se, and phytase 500 FTU.

**Table 3:** Effect of silage supplementation on broiler performance parameters.

Body Weight (g) on Day	Silage-0%	Silage-5%	Silage-10%	SEM	*p*-Value
1	42.0	42.1	42.3	0.081	0.199
15	437.1	439.2	455.1	4.277	0.206
22	842.2 ^ab^	810.8 ^a^	866.2 ^b^	7.135	0.021
35	1605.7 ^a^	1533.6 ^a^	1721.1 ^b^	15.551	0.001
**Weight gain (g) for days**					
1–15	395.1	397.1	412.7	4.275	0.216
15–22	405.1 ^ab^	371.7 ^a^	411.1 ^b^	5.527	0.023
22–35	763.5 ^ab^	722.8 ^a^	854.9 ^b^	16.636	0.016
1–35	1563.7 ^a^	1491.5 ^a^	1678.7 ^b^	15.548	0.001
**Daily feed intake (g) for days**					
1–15	32.9	33.8	32.6	0.334	0.339
15–22	78.3	81.0	81.1	0.822	0.306
22–35	131.8 ^a^	139.1 ^a^	165.1 ^b^	1.828	<0.001
1–35	77.8 ^a^	81.4 ^a^	90.6 ^b^	0.710	<0.001
**FCR ^1^ (g feed/g WG) for days**					
1–15	1.1530	1.1943	1.0891	0.018	0.080
15–22	1.3538 ^a^	1.5296 ^b^	1.3878 ^ab^	0.024	0.019
22–35	2.2571	2.5308	2.5176	0.056	0.116
1–35	1.7379 ^a^	1.9152 ^b^	1.8801 ^b^	0.022	0.011

^a,b^ Means (*n* = 6 per treatment) with no common superscript differ significantly (*p* ≤ 0.05). ^1^ FCR = feed conversion ratio.

**Table 4:** Effect of silage supplementation on broiler intestinal microflora populations.

Jejunum Microbes (Log_10_ CFU/g)	Silage-0%	Silage-5%	Silage-10%	SEM	*p*-Value
Aerobes PCA	5.84	5.62	5.93	0.176	0.770
Anaerobes PCA	7.08 ^a^	7.84 ^b^	8.26 ^b^	0.097	0.001
Enterobacteriaceae	5.78 ^b^	4.89 ^a^	4.50 ^a^	0.105	0.001
Enterococci	6.72 ^b^	4.85 ^a^	5.44 ^a^	0.151	<0.001
Lactobacilli	7.30	7.18	7.63	0.141	0.418
Bifidobacteria	4.73 ^a^	5.67 ^b^	5.99 ^b^	0.145	0.008
**Cecum microbes (Log_10_ CFU/g)**	**Silage-0%**	**Silage-5%**	**Silage-10%**	**SEM**	***p*-value**
Aerobes PCA	8.21 ^ab^	7.70 ^a^	8.49 ^b^	0.117	0.041
Anaerobes PCA	7.77	7.83	8.09	0.122	0.539
Enterobacteriaceae	7.91 ^b^	7.26 ^ab^	6.75 ^a^	0.106	0.002
Enterococci	7.28	7.99	8.10	0.148	0.079
Lactobacilli	7.95 ^a^	7.95 ^a^	8.75 ^b^	0.079	0.001
Bifidobacteria	5.74 ^a^	6.41 ^ab^	6.69 ^b^	0.148	0.050

^a,b^ Means (*n* = 6 per treatment) with no common superscript differ significantly (*p* ≤ 0.05).

**Table 5:** Effect of silage supplementation on broiler blood biochemical parameters.

Blood Parameters ^1^	Silage-0%	Silage-5%	Silage-10%	SEM	*p*-Value
TRIG (mg/dL)	31.67 ^b^	20.83 ^a^	19.08 ^a^	1.443	0.006
ALB (g/dL)	1.13	1.03	1.06	0.034	0.467
ALT (U/L)	22.17	24.08	23.50	1.302	0.829
AST (U/L)	219.58	196.42	210.83	7.117	0.427
CHOL (mg/dL)	74.42 ^b^	57.58 ^ab^	55.08 ^a^	2.669	0.020
TBIL (mg/dL)	0.18	0.13	0.12	0.014	0.232
GLU (mg/dL)	207.75 ^a^	194.83 ^a^	232.50 ^b^	3.255	0.001

^a,b^ Means (*n* = 6 per treatment) with no common superscript differ significantly (*p* ≤ 0.05). ^1^ TRIG: Triglycerides; ALB: Albumine; ALT: Alanine aminotransferase; AST: aspartate aminotransferase; CHOL: Cholesterol; TBIL: Total bilirubin; GLU: Glucose.

**Table 6:** Effect of silage supplementation on broiler breast and thigh meat chemical composition.

Breast Meat Chemical Composition (%)	Silage-0%	Silage-5%	Silage-10%	SEM	*p*-Value
Fat	1.39	1.26	1.38	0.051	0.527
Moisture	74.51	74.78	74.65	0.102	0.568
Protein	23.63	23.34	23.30	0.111	0.435
Collagen	0.78	0.87	0.90	0.029	0.210
Ash	0.75	0.87	0.85	0.023	0.091
**Breast meat color ^1^**					
L*	74.47	72.35	76.14	0.675	0.104
A*	3.97	4.03	4.23	0.161	0.780
B*	2.78 ^b^	0.58 ^a^	3.12 ^b^	0.304	0.008
**Thigh meat chemical composition (%)**	**Silage-0%**	**Silage-5%**	**Silage-10%**	**SEM**	***p*-value**
Fat	4.51	4.17	4.63	0.157	0.479
Moisture	74.66	75.38	75.05	0.147	0.176
Protein	20.52	20.15	20.00	0.129	0.269
Collagen	1.12	1.11	0.97	0.032	0.157
Ash	0.72	0.76	0.74	0.023	0.736
**Thigh meat color ^1^**					
L*	68.52	69.25	71.11	0.626	0.251
A*	7.01	7.44	6.68	0.345	0.668
B*	−0.47	0.25	−0.18	0.475	0.827

^a,b^ Means (*n* = 6 per treatment) with no common superscript differ significantly (*p* ≤ 0.05). ^1^ Lightness (L*), redness (A*) and yellowness (B*) values.

**Table 7:** Effect of silage supplementation on broiler breast and thigh meat microbial populations.

Breast Meat Microbes (Log_10_ CFU/g)	Silage-0%	Silage-5%	Silage-10%	SEM	*p*-Value
Total microbes	6.83 ^b^	6.19 ^ab^	5.91 ^a^	0.138	0.042
*Escherichia coli*	3.19 ^b^	1.77 ^a^	0.99 ^a^	0.193	0.001
*Staphylococcus aureus*	2.93	2.57	2.07	0.148	0.091
*Staphylococcus* spp.	3.72	3.66	3.14	0.120	0.131
*Clostridium* spp.	2.97 ^b^	1.43 ^a^	1.08 ^a^	0.196	0.003
*Campylobacter jejuni*	3.47 ^b^	2.03 ^a^	1.10 ^a^	0.153	<0.001
**Thigh meat microbes (Log_10_ CFU/g)**	**Silage-0%**	**Silage-5%**	**Silage-10%**	**SEM**	***p*-value**
Total microbes	7.13	6.25	6.86	0.140	0.059
*Escherichia coli*	3.90 ^b^	2.22 ^a^	1.76 ^a^	0.252	0.008
*Staphylococcus aureus*	2.80	2.40	2.31	0.157	0.424
*Staphylococcus* spp.	4.06	4.24	4.12	0.213	0.941
*Clostridium* spp.	2.75	2.51	2.34	0.135	0.467
*Campylobacter jejuni*	3.85 ^b^	3.37 ^b^	2.22 ^a^	0.136	0.001

^a,b^ Means (*n* = 6 per treatment) with no common superscript differ significantly (*p* ≤ 0.05).

**Table 8:** Effect of silage supplementation on broiler breast and thigh meat oxidative stability.

Meat MDA ^1^ (ng/g)	Silage-0%	Silage-5%	Silage-10%	SEM	*p*-Value
Breast meat	11.62	17.21	14.67	3.285	0.787
Thigh meat	22.92	20.95	11.40	4.790	0.587
**Meat Phenols (g/L)**	**Silage-0%**	**Silage-5%**	**Silage-10%**	**SEM**	***p*-value**
Breast meat	3.18	3.77	3.73	0.114	0.094
Thigh meat	2.88	3.01	4.02	0.214	0.090
**Meat TBARS ^1^ (mg MDA/kg)**	**Silage-0%**	**Silage-5%**	**Silage-10%**	**SEM**	***p*-value**
Breast meat	0.0510	0.0498	0.0420	0.002	0.107
Thigh meat	0.0826 ^b^	0.0556 ^ab^	0.0554 ^a^	0.004	0.030

^a,b^ Means (*n* = 6 per treatment) with no common superscript differ significantly (*p* ≤ 0.05). ^1^ MDA = malondialdehyde; TBARS = 2-thiobarbituric acid-reactive substances.

**Table 9:** Effect of silage supplementation on broiler breast meat fatty acid composition.

Breast Meat Fatty Acids (%)	Silage-0%	Silage-5%	Silage-10%	SEM	*p*-Value
C14:0 (Myristic)	0.54	0.52	0.46	0.021	0.278
C14:1 (Myristoleic)	0.11 ^b^	0.09 ^b^	0.00 ^a^	0.017	0.001
C15:0 (Pentadecanoic)	0.08	0.08	0.03	0.012	0.146
C16:0 (Palmitic)	25.27 ^b^	25.71 ^b^	24.21 ^a^	0.241	0.003
C16:1 (Palmitoleic)	3.99 ^b^	3.01 ^a^	2.32 ^a^	0.268	0.006
C17:0 (Heptadecanoic)	0.12	0.12	0.12	0.006	0.992
C17:1 (cis-10-Heptadecenoic)	0.04	0.03	0.00	0.008	0.127
C18:0 (Stearic)	6.68 ^a^	8.01 ^ab^	9.51 ^b^	0.456	0.008
C18:1n9t (Elaidic)	0.06 ^a^	0.07 ^a^	0.12 ^b^	0.011	<0.001
C18:1n9c (Oleic)	29.39	27.13	26.32	0.622	0.095
C18:2n6c (Linoleic)	29.02	28.33	28.37	0.394	0.777
C18:3n6 (γ-Linolenic)	0.16 ^b^	0.12 ^a^	0.15 ^ab^	0.007	0.024
C20:0 (Arachidic)	0.09	0.09	0.06	0.010	0.271
C18:3n3 (a-Linolenic)	2.14	1.83	1.84	0.074	0.132
C20:1n9c (cis-11-Eicosenoic)	0.13	0.14	0.16	0.008	0.411
C20:2 (cis-11.14-Eicossadienoic)	0.20 ^a^	0.36 ^ab^	0.50 ^b^	0.051	0.021
C20:3n3 (cis-11.14.17-Eicosatrienoic)	0.24	0.43	0.33	0.059	0.515
C20:4n6 (Arachidonic)	1.41 ^a^	3.25 ^b^	4.54 ^b^	0.486	0.002
C24:1n9 (Nervonic)	0.22 ^a^	0.45 ^ab^	0.53 ^b^	0.054	0.016
C22:6n3 (cis-4.7.10.13.16.19-Docosahexaenoic)	0.09 ^a^	0.23 ^b^	0.24 ^b^	0.029	0.030
Saturated Fatty Acids	32.79	34.53	34.38	0.363	0.070
Unsaturated Fatty Acids	67.19	65.46	65.41	0.395	0.093
Monounsaturated Fatty Acids	33.92 ^b^	30.93 ^ab^	29.45 ^a^	0.802	0.035
Polyunsaturated Fatty Acids	33.27	34.54	35.96	0.646	0.260
n3 (omega-3) Fatty Acids	2.48	2.49	2.41	0.091	0.937
n6 (omega-6) Fatty Acids	30.58	31.70	33.05	0.570	0.225
n6/n3	12.38	12.80	13.98	0.502	0.458

^a,b^ Means (*n* = 6 per treatment) with no common superscript differ significantly (*p* ≤ 0.05).

**Table 10:** Effect of silage supplementation on broiler thigh meat fatty acid composition.

Thigh Meat Fatty Acids (%)	Silage-0%	Silage-5%	Silage-10%	SEM	*p*-Value
C14:0 (Myristic)	0.46	0.47	0.49	0.009	0.422
C14:1 (Myristoleic)	0.06	0.07	0.08	0.006	0.550
C15:0 (Pentadecanoic)	0.05	0.07	0.07	0.008	0.473
C16:0 (Palmitic)	25.24	25.12	23.44	0.375	0.062
C16:1 (Palmitoleic)	2.90	3.19	3.27	0.090	0.227
C17:0 (Heptadecanoic)	0.12	0.13	0.10	0.005	0.059
C17:1 (cis-10-Heptadecenoic)	0.00 ^a^	0.04 ^b^	0.04 ^b^	0.007	0.010
C18:0 (Stearic)	8.54 ^b^	6.85 ^a^	7.25 ^ab^	0.315	0.042
C18:1n9t (Elaidic)	0.09	0.07	0.07	0.006	0.314
C18:1n9c (Oleic)	27.51	29.37	29.73	0.515	0.168
C18:2n6c (Linoleic)	28.74	29.86	30.66	0.496	0.319
C18:3n6 (γ-Linolenic)	0.16	0.13	0.14	0.007	0.125
C20:0 (Arachidic)	0.00 ^a^	0.08 ^b^	0.09 ^b^	0.016	0.006
C18:3n3 (a-Linolenic)	1.84 ^a^	2.22 ^ab^	2.27 ^b^	0.083	0.032
C20:1n9c (cis-11-Eicosenoic)	0.14	0.14	0.13	0.005	0.936
C20:2 (cis-11.14-Eicossadienoic)	0.38 ^b^	0.23 ^a^	0.21 ^a^	0.031	0.019
C20:3n3 (cis-11.14.17-Eicosatrienoic)	0.42 ^b^	0.22 ^a^	0.23 ^a^	0.037	0.016
C20:4n6 (Arachidonic)	2.78 ^b^	1.43 ^a^	1.39 ^a^	0.271	0.025
C24:1n9 (Nervonic)	0.42 ^b^	0.20 ^a^	0.17 ^a^	0.046	0.018
C22:6n3 (cis-4.7.10.13.16.19-Docosahexaenoic)	0.19 ^b^	0.08 ^a^	0.09 ^a^	0.021	0.028
Saturated Fatty Acids	34.41 ^b^	32.72 ^ab^	31.45 ^a^	0.514	0.028
Unsaturated Fatty Acids	65.60 ^a^	67.24 ^ab^	68.48 ^b^	0.500	0.029
Monounsaturated Fatty Acids	31.11	33.08	33.50	0.547	0.165
Polyunsaturated Fatty Acids	34.49	34.17	34.98	0.480	0.827
n3 (omega-3) Fatty Acids	2.44	2.52	2.58	0.051	0.585
n6 (omega-6) Fatty Acids	31.67	31.42	32.19	0.436	0.809
n6/n3	12.96	12.47	12.49	0.154	0.382

^a,b^ Means (*n* = 6 per treatment) with no common superscript differ significantly (*p* ≤ 0.05).

## Data Availability

Not applicable.

## References

[1] Petrotos K., Papaioannou C., Kokkas S., Gkoutsidis P., Skoufos I., Tzora A., Bonos E., Tsinas A., Giavasis I., Mitsagga C. (2021). Optimization of the composition of a novel bioactive silage produced by mixing of ground maize grains with olive mill waste waters, grape pomace and feta cheese whey. AgriEngineering.

[2] Ajila C.M., Brar S.K., Verma M., Tyagi R.D., Godbout S., Valero J.R. (2012). Bio-processing of agro-byproducts to animal feed. Crit. Rev. Biotechnol..

[3] Elwakeel E.A., Titgemeyer E.C., Johnson B.J., Armendariz C.K., Shirley J.E. (2007). Fibrolytic enzymes to increase the nutritive value of dairy feedstuffs. J. Dairy Sci..

[4] Kung L., Shaver R.D., Grant R.J., Schmidt R.J. (2018). Silage review: Interpretation of chemical, microbial, and organoleptic components of silages. J. Dairy Sci..

[5] Kim D.H., Lee K.D., Choi K.C. (2021). Role of LAB in Silage Fermentation: Effect on Nutritional Quality and Organic acid Production-An Overview.

[6] Voidarou C., Antoniadou M., Rozos G., Tzora A., Skoufos I., Varzakas T., Lagiou A., Bezirtzoglou E. (2021). Fermentative foods: Microbiology, biochemistry, potential human health benefits and public health issues. Foods.

[7] Gerasopoulos K., Stagos D., Kokkas S., Petrotos K., Kantas D., Goulas P., Kouretas D. (2015). Feed supplemented with byproducts from olive oil mill wastewater processing increases antioxidant capacity in broiler chickens. Food Chem. Toxicol..

[8] Valeckova E., Ivarsson E., Ellstrom P., Wang H., Kasmaei K.M., Wall H. (2020). Silage and haylage as forage in slow and fast-growing broilers-effects on performance in *Campylobacter jejuni* infected birds. Br. Poult. Sci..

[9] PD (2013). Presidential Degree 56/2013 on Harmonization of the Directive 2010/63/EU, on the Protection of Animals Used for Scientific Purposes.

[10] AOAC (2007). Official Methods of Analysis.

[11] Aviagen (2014). Ross 308 Broiler: Nutrition Specifications.

[12] Premier Nutrition (2014). Premier Atlas 2014. Ingredients Matrix.

[13] NRC (1994). Nutrient Requirements of Poultry.

[14] Yan W., Sun C., Zheng J., Wen C., Ji C., Zhang D., Chen Y., Hou Z., Yang N. (2019). Efficacy of fecal sampling as a gut proxy in the study of chicken gut microbiota. Front. Microbiol..

[15] Lacharme-Lora L., Owen S.V., Blundell R., Canals R., Wenner N., Perez-Sepulveda B., Fond W.Y., Gilroy R., Wigley P., Hinton J.C.D. (2019). The use of chicken and insect infection models to assess the virulence of African *Salmonella* Typhimurium ST313. PLoS Negl. Trop. Dis..

[16] Bujnakova D., Strakova E., Kmet V. (2013). In Vitro evaluation of the safety and probiotic properties of lactobacilli isolated from chicken and calves. Anaerobe.

[17] Dec M., Puchalski A., Urban-Chmiel R., Wernicki A. (2016). 16S-ARDRA and MALDI-TOF mass spectrometry as tools for identification of Lactobacillus bacteria isolated from poultry. BMC Microbiol..

[18] Duskova D., Sedo O., Ksicova K., Zdrahal Z., Karpiskova R. (2012). Identification of lactobacilli isolated from food by genotypic methods and MALDI-TOF MS. Int. J. Food Microbiol..

[19] Adaszynska-Skwirzynska M., Szczerbinska D., Zych S. (2021). The use of lavender (*Lavandula angustifolia*) essential oil as an additive to drinking water for broiler chickens and its In Vitro reaction with enrofloxacin. Animals.

[20] Anderson S. (2007). Determination of fat, moisture, and protein in meat and meat products by using the FOSS FoodScan near-infrared spectrophotometer with FOSS artificial neural network calibration model and associated database: Collaborative study. J. AOAC Int..

[21] Jang A., Liu X.D., Shin M.H., Lee B.D., Lee S.K., Lee J.H., Jo C. (2008). Antioxidative potential of raw breast meat from broiler chicks fed a dietary medicinal herb extract mix. Poult. Sci..

[22] Ahn D.U., Olson D.G., Jo C., Love J., Jin S.K. (1999). Volatiles production and lipid oxidation on irradiated cooked sausage as related to packaging and storage. J. Food Sci..

[23] O’Fallon J.V., Busboom J.R., Nelson M.L., Gaskins C.T. (2007). A direct method for fatty acid methyl ester synthesis: Application to wet meat tissues, oils and feedstuffs. J. Anim. Sci..

[24] Skoufos I., Tzora A., Giannenas I., Bonos E., Papagiannis N., Tsinas A., Christaki E., Florou-Paneri P. (2016). Dietary inclusion of rapeseed meal as soybean meal substitute on growth performance, gut microbiota, oxidative stability and fatty acid profile in growing-fattening pigs. Asian J. Anim. Vet. Adv..

[25] SPSS (2018). SPSS Statistics for Windows, Release 20.0.

[26] Ominski K., McAllister T., Stanford K., Mengistu G., Kebebe E.G., Omonijo F., Cordeiro M., Legesse G., Wittenberg K. (2021). Utilization of by-products and food waste in livestock production systems: A Canadian perspective. Anim. Front..

[27] Svihus B. (2011). The gizzard: Function, influence of diet structure and effects on nutrient availability. World’s Poult. Sci. J..

[28] Steenfeldt S., Kjaer J.B., Engberg R.M. (2007). Effect of feeding silages or carrots as supplements to laying hens on production performance, nutrient digestibility, gut structure, gut microflora and feather pecking behaviour. Br. Poult. Sci..

[29] Wustholz J., Carrasco S., Berger U., Sundrum A., Bellof G. (2016). Silage from alfalfa (*Medicago sativa*) harvested at an early stage as home-grown protein feed for organic broilers. Eur. Poult. Sci..

[30] Tufarelli V., Ragni M., Laudadio V. (2018). Feeding forage in poultry: A promising alternative for the future of production systems. Agriculture.

[31] Voidarou C., Vassos D., Kegos T., Koutsotoli A., Tsiotsias A., Skoufos I., Tzora A., Maipa V., Alexopoulos A., Bezirtzoglou E. (2007). Aerobic and anaerobic microbiology of the immersion chilling procedure during poultry processing. Poult. Sci..

[32] Moretti S., Mrakic-Sposta S., Roncoroni L., Vezzoli A., Dellanoce C., Monguzzi E., Branchi F., Ferretti F., Lombardo V., Duoneda L. (2018). Oxidative stress as a biomarker for monitoring treated celiac disease. Clin. Transl. Gastroenterol..

[33] Rahman M.J., De Camargo A.C., Shahidi F. (2017). Phenolic and polyphenolic profiles of chia seeds and their in vitro biological activities. J. Funct. Foods.

[34] Pappas A.C., Tsiplakou E., Papadomichelakis G., Mitsiopoulou C., Sotirakoglou K., Mpekelis V., Haroutounian S.A., Fegeros K., Zervas G. (2019). Effects of olive pulp addition to broiler diets on performance, selected biochemical parameters and antioxidant enzymes. J. Hell. Vet. Med. Soc..

[35] Barbarestani S.Y., Jazi V., Mohebodini H., Ashayerizadeh A., Shabani A., Toghyani M. (2020). Effects of dietary lavender essential oil on growth performance, intestinal function, and antioxidant status of broiler chickens. Livest. Sci..

[36] Surai P.F. (2014). Polyphenol compounds in the chicken/animal diet: From the past to the future. J. Anim. Physiol. Anim. Nutr..

[37] Sidiropoulou E., Skoufos I., Marugan-Hernandez V., Giannenas I., Bonos E., Aguiar-Martins K., Lazari D., Blake D.P., Tzora A. (2020). In Vitro anticoccidial study of oregano and garlic essential oils and effects on growth performance, fecal oocyst output, and intestinal microbiota In Vivo. Front. Vet. Sci..

[38] Lecci R.M., D’Antuono I., Cardinali A., Garbetta A., Linsalata V., Logrieco A.F., Leone A. (2021). Antioxidant and pro-oxidant capacities as mechanisms of photoprotection of olive polyphenols on uva-damaged human keratinocytes. Molecules.

[39] Makri S., Kafantaris I., Stagos D., Chamokeridou T., Petrotos K., Gerasopoulos K., Mpesios A., Goutzourelas N., Kokkas S., Goulas P. (2017). Novel feed including bioactive compounds from winery wastes improved broilers’ redox status in blood and tissues of vital organs. Food Chem. Toxicol..

[40] Qiao M., Fletcher D.L., Smith D.P., Northcutt J.K. (2001). The effect of broiler breast meat color on pH, moisture, water-holding capacity, and emulsification capacity. Poult. Sci..

[41] Betti M., Perez T.I., Zuidhof M.J., Renema R.A. (2009). Omega-3-enriched broiler meat: 3. Fatty acid distribution between triacylglycerol and phospholipid classes. Poult. Sci..

[42] Storlein T.H., Pan D.A., Kriketos A.D., O’Connor J., Caterson I.D., Cooney G.J., Jenkins A.B., Baur L.A. (1995). Skeletal muscle membrane and storage lipids, muscle fibre type and insulin resistance. Lipids.

[43] Fan R., Kim J., You M., Giraud D., Toney A.M., Shin S.H., Kim S.Y., Borkowski K., Newman J.W., Chung S. (2020). α-Linolenic acid-enriched butter attenuated high fat diet-induced insulin resistance and inflammation by promoting bioconversion of n-3 PUFA and subsequent oxylipin formation. J. Nutr. Biochem..

[44] Hernández-Sánchez J., Amills M., Pena R.N., Mercadé A., Manunza A., Quintanilla R. (2013). Genomic architecture of heritability and genetic correlations for intramuscular and back fat contents in Duroc pigs. J. Anim. Sci..

[45] Gregory M.K., Gibson R.A., Cook-Johnson R.J., Cleland L.G., James M.J. (2011). Elongase reactions as control points in long-chain polyunsaturated fatty acid synthesis. PLoS ONE.

[46] Ogłuszka M., Szostak A., Te Pas M.F.W., Poławska E., Urbański P., Blicharski T., Pareek C.S., Juszczuk-Kubiak E., Dunkelberger J.R., Horbańczuk J.O. (2017). A porcine gluteus medius muscle genome-wide transcriptome analysis: Dietary effects of omega-6 and omega-3 fatty acids on biological mechanisms. Genes Nutr..

